# Comparative Evaluation of Optical Alignment Algorithms for Integrated Probe Cards in Photonic Wafer Testing

**DOI:** 10.3390/mi17050592

**Published:** 2026-05-12

**Authors:** Mehdi Bejani, Alessia Galli, Riccardo Vettori, Marco Mauri, Stefano Mariani

**Affiliations:** 1 Department of Civil and Environmental Engineering, Politecnico di Milano, 20133 Milano, Italy; stefano.mariani@polimi.it; 2 Technoprobe, 23870 Cernusco Lombardone, Italy; alessia.galli@technoprobe.com (A.G.); riccardo.vettori@technoprobe.com (R.V.); marco.mauri@technoprobe.com (M.M.)

**Keywords:** Photonic Integrated Circuits, wafer-level testing, probe card, optical alignment, piezoelectric actuation, Bayesian optimization, hysteresis compensation

## Abstract

Wafer-level testing of Photonic Integrated Circuits (PICs) represents a critical throughput bottleneck in silicon photonics manufacturing, particularly as co-packaged optics demand testing of thousands of optical I/O per wafer. This work introduces optimized alignment algorithms for the Technoprobe Eclipse Dynamic probe card system, which integrates electrical probes and a piezoelectrically actuated fiber array unit within a single probe head, eliminating external positioning equipment. We systematically evaluate seven alignment algorithms: Reference Coarse Scan, Reference Coarse+Fine Scan, Cross Scan, Local and Global Bayesian Optimization, Variable and Fixed Gradient Ascent. The evaluation is made across 72 simulated test cases derived from eight experimental datasets through systematic spatial windowing, combined with experimental validation. Performance is assessed under four operating regimes—high-speed (HS) and low-speed (LS) operation, each with or without hysteresis compensation (H/NH). Experimental validation across eight die positions confirms 100% success rate for both Local Bayesian (98.24% accuracy in 99.87 arbitrary units (a.u.)) and Fixed Gradient (99.18% accuracy in 154.01 a.u.) baseline algorithms. Comprehensive simulation results with improved algorithms across all four scenarios reveal distinct performance characteristics. Fixed Gradient achieves the highest reliability (95.8%) with 99.4% average accuracy across all operating conditions. Variable Gradient provides the fastest alignment (1.18 a.u. in HS-NH) with 90.3% reliability. Local Bayesian demonstrates 94.4% reliability with intermediate performance. Global Bayesian Optimization achieves the best sample efficiency (average 24 steps) but exhibits scenario-dependent reliability ranging from 88.9% (HS-H, LS-H) to 93.1% (LS-NH). For the ideal production scenario, high speed with effective hysteresis compensation (HS-NH), Fixed Gradient emerges as the optimal choice, delivering 95.8% reliability with 1.44 a.u. alignment time, resulting in the best success rate while being nearly as fast as the fastest method. Variable Gradient achieves the absolute fastest alignment (1.18 a.u.) but with 5.5% lower reliability (90.3%), making it suitable only for applications tolerating higher failure rates. Under realistic production conditions with uncompensated hysteresis (HS-H), Fixed Gradient maintains its advantage (95.8% reliability, 3.32 a.u.), while Global Bayesian degrades significantly (88.9% reliability, 4.29 a.u.). Statistical analysis using data profiles validates these methods for high-volume PIC manufacturing, with the Eclipse Dynamic system demonstrating per-die optical alignments in sub-second timescales using open-loop control hardware.

## 1. Introduction

Wafer-level testing of photonic integrated circuits (PICs) has emerged as a critical bottleneck in the silicon photonics manufacturing flow [1]. The convergence of optical and electronic components in PICs has revolutionized high-speed data transmission, telecommunications, and sensing applications [1]. As PIC complexity grows exponentially, driven by demands from data centers and 5G networks, wafer-level testing has become increasingly critical to ensure device functionality and yield before expensive packaging processes [2].

Unlike standard electronic ICs, PICs require simultaneous electrical and optical characterization, presenting unique challenges for test equipment design. A fundamental bottleneck in PIC wafer probing is achieving precise optical coupling between optical fibers and on-chip waveguides, which demands alignment tolerances typically in the range of 100 nm or better across six degrees of freedom [3,4]. Traditionally, these alignments have been performed using external optical positioning systems, such as hexapods and piezoelectric actuators mounted on prober peripheries, or by slow automated routines. While functional in lab settings, these external setups significantly increase equipment cost, footprint, and setup complexity, resulting in substantial test time per device [4]. With testing already accounting for roughly 30% of the total manufacturing cost of a PIC device [3], these external systems severely limit throughput and make parallel testing of multiple devices challenging. This time and cost pressure is only growing as PIC volumes increase and diverse optical I/O designs (grating couplers, edge couplers, etc.) proliferate.

The industry moving toward co-packaged optics (CPO) in high-performance computing and data centers further amplifies the challenge, as wafer-level optical testing must scale to meet the throughput demands of potentially thousands of optical I/O per wafer [1,3]. In CPO-enabled photonic engines, PIC dies with dramatically higher port counts need to be tested rapidly to avoid becoming a production bottleneck [3].

Recent advances have fundamentally changed the architecture of PIC wafer test equipment. On one front, there is a push toward integrated probe head solutions that combine optical and electrical probing in a single apparatus, eliminating the need for separate bulky aligners. One such design philosophy relies on passive, alignment-insensitive optical coupling: the on-card optics are engineered so that the positional tolerances of the prober chuck alone provide adequate coupling, effectively removing per-die active alignment overhead and pushing throughput toward the electrical-probing limit [4,5,6].

Our work is based on the Technoprobe Eclipse Dynamic probe system, an alternative approach which co-integrates a fiber array unit (FAU) and piezoelectric micro-positioners directly into the probe card [7,8]. The FAU is mounted on a flexure mechanism and actuated by three piezoelectric elements operating over a voltage range of 0–120 V, providing a displacement range of 15 μm × 15 μm alongside necessary tilt compensation. This enables high-precision optical alignment through automated routines without the need for external positioning equipment. Rather than relying on cumbersome, large, and slow external tools, this design integrates the active alignment capability directly into a compact, dynamic probe head, drastically reducing move-and-align times between dies. Integration of intelligent control and monitoring frameworks [9] further enhances the capability of the system for high-volume production environments. As a result, the typical active alignment time (which once took on the order of tens of seconds per die) can be significantly reduced [4,10]. This is a necessary evolution for high-volume scenarios: an integrated optical probe card can save precious seconds per die in alignment, which compounded over hundreds of dies yields substantial throughput gains [6,10,11].

Equally important, integrating the optical aligners into the probe hardware enables parallel multi-channel testing. For instance, the Eclipse Dynamic (and similar systems) can couple many fiber channels at once, leveraging parallel optical tests to further boost throughput. Probe-card-based architectures that co-integrate optical and electrical interfaces are already enabling this parallelism [5,12], and recent fully automated wafer-level systems demonstrate the viability of high-throughput multi-site electro-optical characterization [10].

Another key aspect of our solution is its cost-effective control strategy. High-performance aligners often employ closed-loop feedback (using interferometric sensors or strain gauges) to achieve nanometric precision and to counteract piezo hysteresis and drift. Piezoelectric actuators inherently exhibit this hysteresis behavior, where displacement depends not only on the applied voltage but also on the voltage history [13]. Because this non-linearity is particularly pronounced at higher voltages, it can significantly impact alignment accuracy and speed. However, implementing closed-loop control to mitigate this in a dense multi-fiber probe head would add considerable complexity and cost. Instead, the Eclipse Dynamic system exploits advanced open-loop control with algorithmic compensation for these actuator nonlinearities. Furthermore, because each measurement point requires time for actuator movement and signal settling, the choice of alignment algorithm and operating speed regime plays a crucial role in determining overall testing throughput.

However, modern modeling techniques for piezoelectric actuators have made it feasible to achieve accuracies of a few nanometers without physical feedback sensors. Hysteresis operators like the Preisach and Prandtl–Ishlinskii (P-I) model can be mathematically inverted for feedforward linearization of piezo stages [14,15]. Recent research has improved upon these classical models to address rate-dependent and asymmetric hysteresis, using modified P-I operators or even data-driven approaches (e.g., sparse and machine-learning-based hysteresis models) for greater accuracy [16,17]. By leveraging such techniques, our piezo actuators in the probe head are software-linearized, enabling repeatable sub-micron alignments in open-loop. This avoids the need for embedded nano-position encoders on each axis, thereby simplifying the probe hardware and enhancing robustness in a production environment.

In this work, we present a comprehensive study of alignment optimization techniques. We evaluate seven different alignment algorithms through both experimental measurements and simulated data generated from experimental scans. Each algorithm was evaluated across eight distinct on-wafer die locations (spanning multiple reticle positions) directly on the Eclipse Dynamic hardware. Our analysis considers both high-speed (HS) and low-speed (LS) operating regimes, as well as the impact of piezoelectric hysteresis on alignment performance. Following best practices for algorithm comparison [18], we employ data profile analysis rather than simple averaging to account for the non-normal distribution of convergence steps, particularly as the number of optimization problems increases.

The key contributions of this work can thus be listed as:

- Comprehensive experimental characterization of seven alignment algorithms on eight real wafer-level PIC coupling scenarios;
- Simulation framework for generating synthetic alignment scenarios with controllable hysteresis effects;
- Statistical analysis using data profiles for rigorous algorithm comparison;
- Comparative performance evaluation across high-speed and low-speed operating conditions;
- Practical guidelines for algorithm selection based on operating regime and hysteresis conditions;
- Systematic hyperparameter optimization of Bayesian algorithms between experimental and simulation phases.

While the individual search algorithms utilized in this study are established numerical methods, the core contribution of this work is their rigorous, system-level benchmarking within the strict hardware constraints of industrial PIC testing. By evaluating these algorithms under diverse operational extremes, we provide actionable guidelines for production deployment. These algorithms are particularly vital for emerging coupling approaches, such as 3D-printed edge couplers or micro-optics [19], where device-to-device spatial variations are larger and demand highly robust dynamic active alignment.

The remainder of this paper is organized as follows: Section 2 reviews related work in PIC testing and alignment optimization. Section 3 describes the Eclipse Dynamic system and details the alignment algorithms under investigation. Section 4 presents experimental and simulation results with data profile analysis. Section 5 discusses the comparative performance and provides recommendations. Finally, Section 6 concludes the paper and outlines future work.

## 2. Background

### 2.1. Wafer-Level Photonic Testing and Integrated Probe Head Architectures

Wafer-level testing of PICs has attracted growing attention, driven by the need to validate optical performance at scale before costly packaging steps [3,4]. A central challenge is related to achieving reliable, repeatable fiber-to-chip optical coupling across hundreds of dies per wafer, each requiring sub-micron positional accuracy in multiple degrees of freedom [3]. Early test setups relied on ad-hoc laboratory configurations in which skilled operators manually aligned individual optical fibers using bench-top micro-positioners, resulting in test times on the order of minutes per die and making high-volume characterization practically infeasible [4]. The broader silicon photonics community has since worked toward automated, reproducible measurement flows that integrate optical probing with standard wafer probers [2,4].

A key architectural trend is the consolidation of optical and electrical probing into a unified probe head compatible with conventional wafer probers, eliminating the need for separate, bulky fiber positioning equipments. Two distinct philosophies have emerged. The first relies on passive, alignment-insensitive optical coupling: by carefully engineering the beam characteristics of the on-card optics, the positional tolerances of the prober chuck become sufficient for adequate coupling without any per-die active correction, effectively removing per-die alignment overhead and pushing throughput toward the electrical-probing limit [4,5]. The second philosophy, adopted in the present work, retains active micro-positioning of the FAU within the probe head using compact piezoelectric actuators, allowing fine coupling optimization on each die and accommodating device-to-device variation at the cost of a short, software-driven alignment routine per die [7,8]. Both approaches represent a clear departure from external hexapod or gantry aligners attached to the prober periphery, which added equipment cost, floor-space overhead, and limited the degree of test parallelism achievable.

Probe-card-based solutions capable of parallel multi-channel measurements have been demonstrated for a range of PIC types. Petrini et al. integrated a fiber-array probe with an electrical card to enable simultaneous multi-channel characterization of photonic filters without external positioning equipment [5]. Wu et al. demonstrated a butt-coupling probe card with on-board polarization control, allowing polarization-sensitive PICs to be tested at wafer scale without any separate fiber aligner [12]. Recent work by Jansen et al. presented a systematic optimization of optical alignment routines specific for high-throughput PIC testing, confirming that algorithm selection has a direct and quantifiable impact on test throughput and yield in production environments [11]. Fully automated edge-coupling probers have further shown that optimized optical search routines and move sequences can reduce per-die alignment time to the multi-second range, yielding throughputs on the order of several thousand optical alignments per hour in single-fiber scenarios [10].

A complementary challenge, intimately linked to both alignment speed and precision, concerns the motion stage itself. In automated PIC assembly and test, the demands for high positioning precision and high throughput are fundamentally opposed: operating a motion stage faster imposes higher control bandwidth requirements and degrades repeatability [20]. Mandelli et al. developed machine-learning models to predict motion stage precision as a function of operating speed for PIC assembly systems, demonstrating that data-driven approaches can characterize and compensate for these trade-offs without offline calibration runs [20]. These findings directly motivate the evaluation of high-speed operating regimes in the present work, and the need to understand how alignment algorithms degrade under reduced repeatability conditions.

### 2.2. Optical Coupling Methods

**Figure 1:**
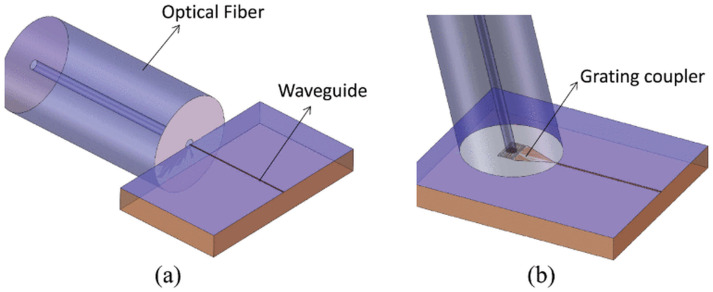
Schematic diagrams of the two primary optical coupling approaches: (**a**) traditional horizontal edge coupling (which, for high-density wafer-level testing, is modified using micro-lenses to allow vertical fiber access) and (**b**) Grating coupling [26].

Two primary optical coupling architectures are relevant to wafer-level PIC testing, as illustrated in Figure 1. As a foundational concept, edge couplers offer high coupling efficiency and broad optical bandwidth by laterally aligning an optical fiber to the cleaved or polished chip edge, requiring tight positional tolerances (typically below 1 μm) [21,22]. However, as illustrated conceptually in Figure 1a, pure lateral access is often physically impossible in densely packed wafer-scale environments, necessitating the use of specialized vertical-to-horizontal beam delivery tools. Grating couplers provide a more convenient vertical-incidence interface compatible with full-wafer probing without edge preparation, but at the expense of higher insertion loss, narrower wavelength bandwidth, and moderate polarization sensitivity [23,24].

Most integrated probe card solutions for grating couplers rely either on external positioning stages or on beam-expansion optics to reduce sensitivity to lateral misalignment, the latter incurring an additional insertion-loss penalty [25]. The Eclipse Dynamic system investigated here targets grating coupler interfaces, where the piezoelectric FAU provides the fine positioning needed to maximize coupling efficiency across the full 15μm×15μm scan range.

### 2.3. Alignment Optimization Algorithms

The selection of the optical alignment search algorithm fundamentally determines both the speed and the reliability of the per-die coupling step in photonic testing. Conventional procedures have historically relied on brute-force scanning or iterative hill-climbing techniques to locate the optimal coupling position. A typical approach involves raster or spiral scanning, where the optical fiber is translated exhaustively across a multidimensional grid until an initial optical signal is detected, followed by dense sweeps to map the coupling efficiency landscape and identify the peak. Although robust to complex landscape topologies, these exhaustive methods require a prohibitively large number of actuator motions and power readings. Consequently, they can consume dozens of seconds for a single alignment, making them impractical for high-volume manufacturing scenarios, especially when multiple degrees of freedom are involved [4]. To mitigate this measurement burden, cross-scan or dimensional-reduction approaches sequentially sweep along individual axes to identify approximate peak coordinates before performing a focused local refinement scan.

To further accelerate the alignment process, modern systems increasingly employ advanced optimization algorithms, such as gradient-based searches. These methods estimate local coupling gradients, often through small dither motions, and steer the optical array in the ascent direction to rapidly approach the optimum. Analogous to numerical gradient descent or ascent, this targeted approach can converge significantly faster than blind scanning. For instance, substituting a coarse spiral scan with an optimized gradient search has been shown to yield an approximate 5× reduction in fiber coupling time [10]. Despite their simplicity and speed, gradient methods are inherently susceptible to local maxima in the measured optical power and require careful tuning of step-size parameters, particularly when the coupling landscape contains secondary lobes or is distorted by actuator nonlinearities [10,11]. A central tension in these systems lies in balancing aggressive, adaptive step sizes that yield the fastest convergence on benign landscapes against conservative, fixed step sizes that maintain reliability on more challenging topologies.

As a powerful alternative, Bayesian optimization (BO) utilizing Gaussian Process (GP) surrogates has emerged to address the limitations of both grid-based and gradient methods. This approach treats the coupling yield as an unknown black-box function, building a probabilistic surrogate model that is iteratively updated with each measurement [27]. By intelligently maximizing an acquisition function such as Expected Improvement, the optimizer strategically balances the exploration of uncertain regions against the exploitation of known high-power areas. This enables the algorithm to infer the global landscape shape and locate optima using far fewer evaluations than traditional methods. Originally popularized in complex domains like X-ray beamline autoalignment [28] and online control of particle accelerators [29], BO excels in environments where the objective function is expensive to evaluate and subject to noise. These characteristics are highly analogous to optical power measurements affected by piezoelectric nonlinearities and mechanical vibrations. Machine-learning-driven alignment has similarly demonstrated success in multidimensional free-space optical systems and lens assemblies, proving the efficacy of Bayesian algorithms in handling complex alignments with minimal iterations [30].

Within the BO family, two distinct paradigms are particularly relevant to the photonic alignment problem. Global BO explores the entire search space from the outset, relying purely on the GP posterior to select the next sampling location. While this maximizes coverage and sample efficiency, it scales poorly with search-space dimensionality and can be sensitive to landscape distortions introduced by actuator nonlinearities. Conversely, local BO restricts the search to a neighborhood around a promising region identified by an initial coarse scan. This restricted approach sidesteps the curse of dimensionality and delivers strong empirical performance while maintaining rigorous convergence guarantees [31]. Theoretical analyses have characterized the asymptotic behavior of local BO, confirming conditions under which locally found stationary points closely approximate the global optimum [31]. Furthermore, replacing standard gradient-descent exploitation with a strategy that minimizes the upper confidence bound over the GP posterior yields tighter theoretical guarantees and improved empirical results [32]. These theoretical advances directly motivate localized Bayesian strategies, which combine a coarse cross-scan initialization with a focused GP refinement step.

Beyond the optimization of single-channel algorithms, the transition toward parallel, multi-channel alignment represents a critical advancement for wafer-level testing. Because modern integrated probe architectures can simultaneously engage multiple input and output fiber pairs across a die, applying alignment algorithms concurrently to all channels drastically shortens the overall test time. Aligning channels sequentially is highly inefficient; instead, coordinated multi-dimensional searches that optimize all optical ports together are essential for maximizing throughput. Ultimately, the field is rapidly transitioning from simplistic single-variable scans toward intelligent, multi-variable, and machine-learning-augmented optimizations. Through the integration of these advanced algorithmic approaches, the optical alignment step can be reduced to a minor fraction of the total test time, making active alignment on the scale of a few seconds a viable reality for high-volume production.

### 2.4. Hysteresis and Nonlinearity Compensation

Piezoelectric actuators are extensively utilized in nanometric alignment systems due to their high resolution and rapid response times. However, a persistent challenge is their inherent hysteresis and creep. The displacement produced by the actuator at a given drive voltage depends not only on that specific voltage but on the entire preceding voltage history, a phenomenon driven by domain-wall motion and polarization switching within the piezoelectric ceramic [13]. In open-loop operation, this path-dependent nonlinearity typically introduces positioning errors spanning 10–15% of the total travel range [33]. Such substantial deviations far exceed the strict sub-micron tolerances required for reliable fiber-to-waveguide alignment in PICs.

The traditional remedy for addressing hysteresis is closed-loop control. By integrating an embedded feedback sensor such as a capacitive sensor, strain gauge, or linear variable differential transformer (LVDT), and utilizing PID or iterative control, systems can dynamically correct positioning errors to achieve sub-nanometer accuracy. While highly effective, closed-loop architectures introduce significant drawbacks: they are more expensive, impose bandwidth limitations, and add substantial hardware complexity. For dense, multi-channel probe heads (e.g., a 16-channel array), embedding a dedicated physical sensor and its associated wiring on every piezoelectric axis is highly impractical. Position sensors, moreover, require space for installation that is not available in the application hereby discussed.

To circumvent these hardware limitations, the preferred strategy is open-loop control enhanced by software-based feedforward compensation algorithms. By characterizing the hysteresis behavior of the piezo and applying an inverse model to the drive signals, the response of the actuator can be effectively linearized. This software-driven approach scales effortlessly across several channels without the prohibitive overhead of extra hardware. Over the past two decades, various frameworks have been developed to model and compensate for piezoelectric hysteresis. Classical approaches include the Preisach model, which captures hysteresis through a superposition of elementary bistable operators, and the P-I model, which employs a weighted sum of play operators [14,15]. Conceptually, a play operator acts as a mathematical representation of mechanical backlash: its output remains constant upon a reversal of direction until the input change exceeds a defined threshold width, after which the output linearly tracks the input. The P-I model is particularly notable because it admits an analytical inverse that can be cascaded with the physical actuator as a feedforward pre-distortion signal to yield a linear response.

To handle more complex behaviors like asymmetric hysteresis, saturation, and rate dependence, advanced direct-inverse and modified approaches have been developed [16,34]. For example, classical symmetric P-I play operators, which apply the exact same threshold and slope regardless of whether the voltage is increasing or decreasing, struggle to capture the divergent ascending and descending branches of physical piezoceramics. Utilizing modified asymmetric play operators, which assign distinct mathematical characteristics to the forward and return paths, allows for a more accurate description of this directional behavior with fewer model parameters [35]. Furthermore, while the classical P-I model possesses a straightforward analytical inverse, utilizing the aforementioned modified models often introduces complex mathematical inversions that are computationally heavy for real-time control. To resolve this, Gu et al. demonstrated a direct-inverse compensation method that characterizes the inverse hysteresis effect directly, bypassing these heavy calculations and achieving tracking error reductions of up to 90% [36]. Similarly, Yu et al. utilized a rate-dependent modified P-I model to reduce linear positioning errors from 10% to below 1% [33].

More recently, data-driven methods have emerged as a powerful alternative. Machine learning approaches, such as the sparse system identification method proposed by Chandra et al., can discover hysteresis models for specific piezo materials, demonstrating residual strain errors well below 1%, which stands as a performance that rivals closed-loop systems [17]. Ultimately, the viability of these various feedforward P-I linearization techniques has been successfully validated in multi-axis optical alignment stages [37,38]. In our implemented system, we calibrate each piezo axis using a variant of the P-I model. By applying a series of voltage trajectories and recording the displacement via an external laser sensor during the calibration phase, we identify the exact hysteresis curve. This model is then inverted in real time by the controller to pre-distort commanded moves. Additionally, a drift compensation table is applied via software to administer small, time-based offset adjustments that counteract the slow creep occurring after a step maneuver.

Crucially, the impact of hysteresis extends beyond spatial accuracy; it introduces a significant temporal penalty. When an alignment algorithm commands a move to a lower voltage position, the system must perform a reset-to-zero maneuver to ensure the actuator remains on the well-characterized forward path, rather than traversing an unpredictable return path. This reset requirement fundamentally differentiates the movement overhead of various search algorithms. Methods that frequently require downward voltage moves, such as Bayesian algorithms that prioritize broad exploration before exploitation, incur a disproportionate time penalty compared to monotone hill-climbing strategies. Understanding how this temporal penalty interacts with the specific search strategy of each algorithm across different operating speeds is a key contribution of this work. As our results in Section 4 will demonstrate, this carefully implemented open-loop strategy reliably aligns fibers with optical loss repeatability greater than 0.1 dB, validating that an algorithmic approach to hysteresis can successfully meet the nanometric tolerances of high-volume wafer-level photonic testing.

### 2.5. Algorithm Comparison Methodology

Traditional algorithm comparison often relies on averaging performance metrics such as number of function evaluations or convergence time. However, as noted by Beiranvand et al. [18], this approach can be misleading when the distribution of performance metrics is non-normal, particularly as the number of optimization problems increases. They advocate for more rigorous statistical approaches including performance profiles and data profiles.

Performance profiles [39] measure the fraction of problems solved within a given performance ratio relative to the best solver. Data profiles [40] extend this concept by plotting the fraction of problems solved as a function of the number of function evaluations, providing insight into convergence speed and robustness. These profile-based methods are particularly appropriate for optimization algorithm comparison, as they account for the full distribution of performance rather than relying on potentially misleading averages.

## 3. Methodology

### 3.1. Eclipse Dynamic System Architecture

To address the severe spatial constraints associated with conventional lateral edge coupling in high-density wafer-level environments, the Eclipse Dynamic system employs lensed fibers and 3D-printed micro-lenses [19] to redirect the optical signal from horizontal propagation to vertical access. This configuration enables dense wafer-level probing while remaining compatible with standard prober geometries and integrated electro-optical test operation.

**Figure 2:**
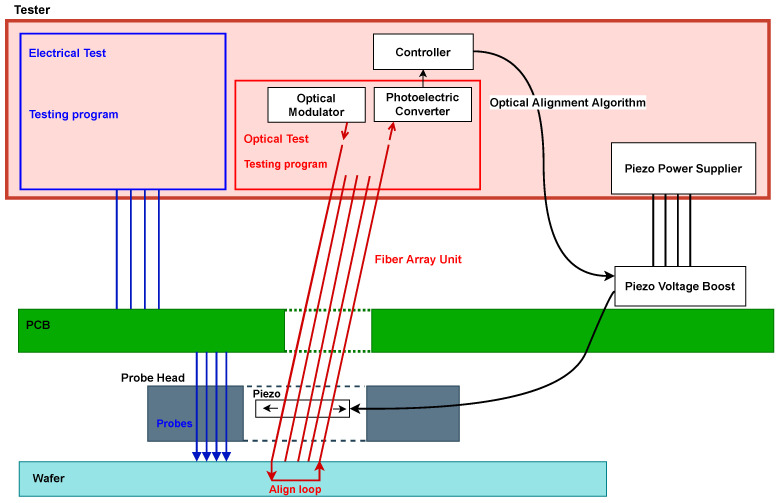
System-level block diagram of the automated wafer-level photonic testing platform, showing the tester, controller, optical source/detection chain, piezoelectric drive electronics, integrated probe head, and wafer under test.

Figure 2 presents the system-level architecture of the Eclipse Dynamic probe card during wafer-level testing. The tester provides both the electrical test program and the optical test program. The optical path comprises a source/modulation stage and a detection stage connected to the integrated FAU in the probe head, while the mechanical alignment loop is closed through the controller and the piezoelectric drive electronics. The FAU is integrated into the probe head together with the electrical probes, such that optical and electrical contact are established on the same wafer site during probing. Rather than actuating individual fibers independently, the system displaces the entire FAU as a single rigid body in the X–Y plane, with differential actuation used for tilt compensation. This architecture eliminates the need for external optical positioning stages and enables compact, high-throughput wafer-level electro-optical testing.

**Figure 3:**
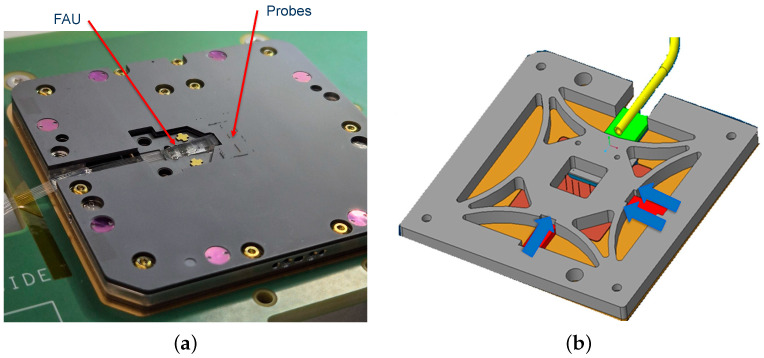
Eclipse Dynamic Probe Head architecture. (**a**) Complete probe head assembly showing electrical probes and FAU integration. (**b**) Internal flexure mechanism showing the piezoelectric actuators (X, Y1, Y2) used for nanometric alignment, with their respective positions marked in blue [8].

Structurally, the probe head consists of two ceramic layers with guiding holes for the electrical probes, separated by a metallic flexure structure, as illustrated in Figure 3. Based on the patented mechanical architecture [41], this flexure supports the integrated FAU and is actuated by three piezoelectric elements: one actuator controls the X-axis, while two parallel actuators (Y1 and Y2) control the Y-axis. Differential driving of Y1 and Y2 provides the rotational tilt compensation required for precise optical alignment, whereas the vertical Z-axis approach is handled by the prober chuck rather than by the integrated piezoelectric stage.

The embedded FAU is a configurable interface rather than a fixed-format optical array. In particular, the number of fibers and the fiber pitch are selected according to the PIC under test, including the number of optical channels, the spacing between input/output ports, and the pitch of the access couplers or waveguides on the chip. Accordingly, the FAU geometry is customized to match the DUT layout while remaining compatible with the mechanical and electrical constraints of the integrated probe head. More generally, the underlying probe-head concept supports multi-channel optical interfacing through an optical distribution element comprising a plurality of waveguides aligned to the probe-head structure by dedicated alignment features [41]. Because the input and output fibers are fixed within one rigid FAU block, they are aligned simultaneously as a unified assembly rather than sequentially.

The actuators are controlled through the probe card PCB, driven by the tester system. The operating voltage range is 0–120 V, corresponding to a total displacement range of 15 μm × 15 μm in the X–Y plane. Under typical operating conditions, the system achieves positioning resolution better than 1 nm, enabling precise alignment to grating couplers with typical mode-field diameters of 10–15 μm.

Because the probe head is a highly compact integrated electro-optical environment, embedding physical displacement sensors for closed-loop optical alignment would introduce undesirable spatial and packaging complexity. Therefore, the system relies on open-loop control. Although the complete hardware architecture and containment-element kinematics have been defined in foundational patents and prior literature [7,8,41], the remainder of this study focuses specifically on the computational efficiency, reliability, and optimization of the automated optical alignment algorithms.

### 3.2. Experimental Characterization

#### 3.2.1. Hysteresis Measurement

As described in Section 2, the piezoelectric actuators in the Eclipse Dynamic system exhibit path-dependent hysteresis whose magnitude scales with the applied voltage range. To quantify this behavior for the specific axes and operating conditions of this work, we performed controlled voltage sweeps on the physical hardware while measuring displacement with a high-resolution optical measurement system. A ceramic reference plate with precision markers was mounted on the flexure, and displacement was recorded as voltage was increased and decreased in steps.

The experimental protocol reads as follows. Voltage was swept for each of the three physical actuators (X, Y1, and Y2) from 0 V to each of six peak levels (20, 40, 60, 80, 100, and 120 V) and returned to 0 V, with the resulting spatial displacement recorded at every step. This protocol isolates how hysteresis error scales with the peak operating voltage.

**Figure 4:**
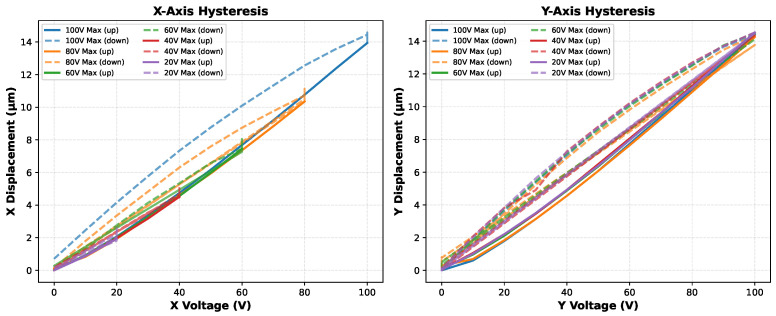
Measured hysteresis loops for Eclipse Dynamic system at different maximum voltages. Hysteresis increases with voltage magnitude, reaching ∼15% at 120 V operation. Solid lines show forward path (increasing voltage), dashed lines show return path (decreasing voltage). **Left**: X-axis behavior. **Right**: Y-axis behavior.

The measured hysteresis loops representing the resulting spatial movement along the X and Y axes are shown in Figure 4. Three characteristics are directly relevant to the algorithm design. First, hysteresis is strongly voltage-dependent: at a peak drive of 100 V the X-axis positioning error is approximately 3.5%, whereas extending to the full 120 V operating range raises this to approximately 15%. Second, the forward path (increasing voltage, solid lines) and return path (decreasing voltage, dashed lines) diverge substantially, with the descending trajectory consistently underreaching the forward-path displacement at equivalent drive levels. Third, restricting actuation to the 20–40 V range limits hysteresis to 2–4%, indicating that algorithms operating via small, localized voltage increments will inherently encounter fewer nonlinearity effects than those requiring wide-range excursions. We additionally observed a slight non-orthogonality between the X and Y axes, attributed to asymmetric Y1/Y2 actuator characteristics and their sequential rather than simultaneous activation; this cross-coupling is accounted for in all alignment algorithm implementations.

These measurements provided the voltage trajectory data used to identify the inverse P-I calibration described in Section 2. The resulting reset-to-zero timing overhead is captured in the hardware timing model of Section 3.3.4.

#### 3.2.2. Optical Power Measurements

The experimental optical setup is configured to measure insertion loss continuously during the active alignment process. The apparatus utilizes a continuous-wave laser source coupled into the single-mode optical fibers of the integrated probe head. Light is injected into the photonic integrated circuit via an input grating coupler, propagates through a dedicated on-die optical loopback waveguide, and is subsequently extracted through an adjacent output grating coupler. The transmitted optical signal is collected by a return fiber within the array and recorded by a high-speed optical power meter. This continuous data stream provides the real-time optical power feedback required by the control software to evaluate coupling efficiency and direct the piezoelectric actuators.

Utilizing this loopback configuration, eight distinct experimental alignment campaigns were conducted across various grating coupler positions and individual dies. This experimental design comprehensively captures the variability arising from die-to-die process fluctuations and position-dependent spatial non-uniformities across the wafer. Furthermore, repeated measurements on a single die provide critical insight into overall system repeatability and environmental stability. Each experimental campaign thus serves as a highly realistic test case for evaluating algorithm robustness under authentic production conditions.

The initial physical measurements were executed using baseline algorithm parameters. During this phase, the BO algorithms employed conservative settings: the Global Bayesian method used 8 initial Latin-Hypercube sampled points [42] and a maximum of 70 iterations, while gradient-based methods used moderate, non-adaptive step sizes and learning rates. These initial parameters, while functional, were not yet optimized for the specific characteristics of the Eclipse Dynamic system or the optical coupling landscape topology observed in practice. The experimental results presented in Section 4 therefore reflect this initial algorithm configuration.

In the updated version of the alignment algorithms used for the comprehensive simulation study, the methods were refined to maximize both efficiency and reliability. Specifically, the gradient-based approaches were upgraded with adaptive step sizes and finer convergence logic to better leverage the system 1 nm hardware positioning resolution. All detailed mathematical parameters and control logic for these refined algorithms, including the specific adaptive formulations, are fully explained in Section 3.4. The simulation framework and the definitive results reported in Section 4 employ these refined, production-ready configurations. By testing these optimized algorithms across 72 unique spatial windows representing a 9× increase in scenario diversity compared to the initial experiments, we established the final performance rankings and recommendations for high-volume PIC manufacturing.

### 3.3. Simulation Framework

**Figure 5:**
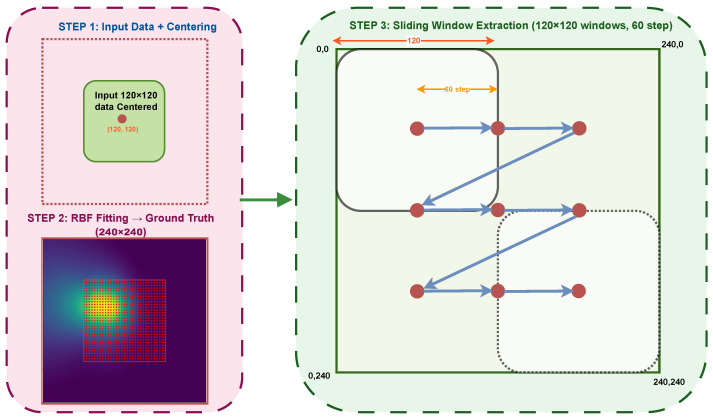
Simulation workflow used to generate synthetic alignment scenarios from experimental scans and to evaluate alignment algorithms under different operating regimes. Experimental data are reconstructed into a high-resolution coupling surface, windowed into multiple mock scenarios, and then replayed through the hardware timing model (with/without reset-to-zero hysteresis strategy) to produce comparable metrics across the four scenarios.

To enable systematic evaluation across a wider range of conditions, we developed a simulation framework that generates synthetic alignment scenarios from experimental data. The objective is to validate algorithm performance without requiring physical hardware by creating high-resolution ground-truth surfaces from discrete measurement data, and generating mock data files that mimic the limited field of view of the hardware during operation. Figure 5 summarizes the simulation pipeline used in this work. Starting from each experimental scan, we reconstruct a continuous ground-truth coupling surface via Radial Basis Function (RBF) interpolation, to establish an extrapolated 240 V × 240 V virtual domain, from which we generate multiple mock scenarios by extracting 120 V × 120 V sub-windows at systematic offsets. For each mock scenario, the alignment algorithms are executed and their recorded trajectories are converted to alignment time using the hardware timing model. This approach allows for direct comparison across four distinct operating regimes without requiring additional wafer measurements: low-speed (LS) and high-speed (HS) actuation, each evaluated with (H) and without (NH) hysteresis compensation, to provide LS-H, HS-H, LS-NH, and HS-NH scenarios.

The framework has three main components, as described in the following.

#### 3.3.1. Surface Reconstruction and Centering

Starting from experimental scan data, we use RBF interpolation with multiquadric kernel to create smooth coupling surfaces. The process consists of the following critical steps.

1. Centering Strategy: The simulation standardizes the input data before fitting. It computes the geometric center of the raw input scan and shifts the data so that it aligns with the center (120, 120) of the global simulation grid. This ensures that the peak is always accessible and not cut off at the edges of the simulation environment.
2. Normalization: To ensure numerical stability during interpolation, the coordinates are temporarily normalized to a 0–1 range.
3. RBF Fitting: We utilize SciPy RBF interpolation function [43] with a multiquadric kernel and smoothing factor (ϵ=0.01) to create a mathematical surface:
  (1) $$P\left(x,y\right)=\sum_{i=1}^{N}w_{i}\phi (∥\left[x,y\right]-\left[x_{i},y_{i}\right]∥)$$
  where $\phi \left(r\right)=\sqrt{r^{2}+\epsilon^{2}}$ is the multiquadric basis function, $w_{i}$ are interpolation weights determined from experimental data points $\left(x_{i},y_{i},P_{i}\right)$, and ϵ is the smoothing parameter.
4. Grid Generation: A dense 240 × 240 pixel grid is generated, representing the ground-truth power values for every possible coordinate in the simulation area. This creates a continuous surface from the discrete experimental measurements.

**Figure 6:**
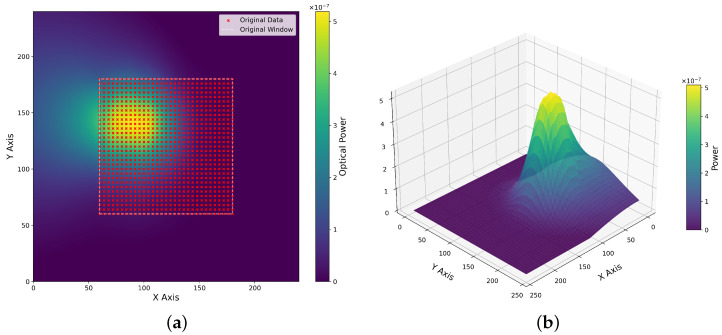
Reconstructed optical power surface from experimental data. (**a**) 2D heatmap showing the 240 × 240 global grid with centered experimental data points (red markers within dashed rectangle). (**b**) 3D surface visualization showing the smooth RBF interpolation and peak power region. The surface extends beyond the measured region to create multiple mock test scenarios.

Figure 6 illustrates this surface reconstruction process. The heatmap (a) shows the interpolated power landscape on the 240 × 240 global grid, with the original experimental data points (red markers) centered at (120, 120) and the dashed rectangle indicating the original 120 × 120 measurement window. The 3D surface plot (b) provides a three-dimensional visualization of the optical coupling landscape, clearly showing the peak power region and the smooth interpolation that extends beyond the measured data points.

#### 3.3.2. Environmental Simulation (Mock Scan Generation)

Real alignment hardware has a limited field of view corresponding to the piezoelectric actuator range (120 V, equivalent to 15 μm displacement), while the simulated surface is 240 units wide. To test algorithm robustness, the large surface is divided into smaller, realistic views using a systematic sliding window technique, according to the following.

1. Sliding Window Process: The sliding window process employs a fixed window size of 120 × 120 units, which corresponds directly to the typical 0–120 V operating range of the piezoelectric stage. To systematically evaluate the data, this window is translated across the global 240 × 240 data matrix using a discrete step size of 60 units. Originating at the (0, 0) coordinate of the global grid, the window slides through sequential positions, such as (0, 60), (0, 120), and (60, 0), ensuring comprehensive and overlapping coverage of the entire surface area in a structured grid pattern.
2. Coordinate Localization: As the window moves to different positions (e.g., Global X = 60 to 180), the system transforms the extracted data back to local coordinates (0–120 V) by subtracting the offset from the window origin. This is critical, as it ensures that the algorithms perceive every file as a standard 0–120 V hardware range, indistinguishable from real operation.
3. Mock File Generation: This systematic windowing process generates 9 distinct mock CSV files from each experimental dataset. Each file contains only the X, Y, and Power data visible within that specific 120 V × 120 V window at offsets of 0, 60, and 120 V in both dimensions. This forces the algorithms to locate the peak in various contexts (e.g., peak centered, peak at the edge, or low-power regions), providing a rigorous test of algorithm robustness across diverse initial conditions.

To provide a statistically rigorous benchmark, a comprehensive simulation library was constructed from the 8 experimental datasets. Each experimental scan was interpolated onto a 240 × 240 voltage grid using a multiquadric RBF to serve as a ground-truth coupling landscape. From these, a total of 72 unique test scenarios (8 experiments × 9 systematic spatial offsets) were generated by extracting 120 V × 120 V sub-windows at varying spatial positions (offsets of 0, 60, and 120 V in each dimension). This systematic procedure ensures that the algorithms are evaluated against a diverse set of initial peak positions relative to the scan window, simulating a wide range of initial fiber-to-chip misalignments that might occur in production testing.

#### 3.3.3. Algorithm Verification

A Headless Aligner script replicates the logic of the main controller. It loads each of the 9 mock files as a virtual device (simulating slew rates and settling times) and runs all 7 alignment strategies sequentially. For every single one of the 9 mock files, the simulation generates 7 result files, one for each algorithm, totaling 63 result files per experimental dataset.

Each generated result file provides a comprehensive record of the alignment procedure. Specifically, it includes a detailed path log that tracks every X and Y coordinate step executed by the algorithm, alongside the corresponding simulated optical power measured at each discrete position. Furthermore, the file incorporates a concluding summary that details the total number of measurement points, the final maximum optical power achieved, and the total execution time. This total alignment time is rigorously calculated by factoring in a simulated hardware slew rate of 100 V/s combined with a constant mechanical settling buffer. To ensure the comparison focuses strictly on algorithmic efficiency rather than hardware-specific speeds, all alignment times throughout this work are expressed in arbitrary units (a.u.). For this normalization, the baseline execution time of the Local BO method under the original LS-H configuration is defined as exactly 100.0 a.u. By comparing these files across all mock scenarios, we can quantify which algorithm is the fastest and most accurate for diverse device profiles.

#### 3.3.4. Hardware Simulation

The simulated hardware model is designed to accurately mimic the mechanical and electrical behavior of the physical alignment system across several key operational parameters. As already discussed, to realistically capture movement timing, the model incorporates two distinct regimes: a HS (high-speed) operation characterized by a rapid slew rate and abbreviated settling times, and a LS (low-speed) operation that employs a more conservative slew rate alongside extended settling durations. Furthermore, the model strictly enforces physical position limits, constraining the simulated actuator travel to a bounded 0–15 μm range (corresponding to a 0–120 V drive signal) across both the X and Y axes. Finally, to simulate dynamic feedback, optical power measurements at any given coordinate are continuously evaluated via direct interpolation from the previously constructed RBF surface, ensuring realistic continuous domain responses during the virtual alignment process.

For hysteresis modeling, when the target position is lower than the current position in either axis, the system simulates a reset-to-zero followed by a move-to-target, approximating the piezoelectric hysteresis behavior according to:

(2) $$t_{\mathrm{move}}=\left\{\begin{array}{cc} \frac{d_{\mathrm{reset}}}{v}+\frac{d_{\mathrm{target}}}{v}+2t_{\mathrm{settle}} & \mathrm{if}\;x_{\mathrm{target}}<x_{\mathrm{current}}\;\mathrm{or}\;y_{\mathrm{target}}<y_{\mathrm{current}}\\ \frac{d_{\mathrm{direct}}}{v}+t_{\mathrm{settle}} & \mathrm{otherwise} \end{array}\right.$$

where the voltage distance terms are defined as:

(3) $$\begin{array}{cc} d_{\mathrm{reset}} & =\sqrt{x_{\mathrm{current}}^{2}+y_{\mathrm{current}}^{2}}\\ d_{\mathrm{target}} & =\sqrt{x_{\mathrm{target}}^{2}+y_{\mathrm{target}}^{2}}\\ d_{\mathrm{direct}} & =\sqrt{{\left(x_{\mathrm{target}}-x_{\mathrm{current}}\right)}^{2}+{\left(y_{\mathrm{target}}-y_{\mathrm{current}}\right)}^{2}} \end{array}$$

with *v* representing the effective slew rate and $t_{\mathrm{settle}}$ the settling time.

This reset-to-zero strategy ensures that when moving to a lower voltage position, the system first returns to the origin (0, 0) and then moves to the target position along the forward (increasing voltage) path. This approach avoids the unpredictable displacement that would occur if the system attempted to follow the return (decreasing voltage) hysteresis path, thereby ensuring repeatable positioning at the cost of additional movement time. To evaluate the absolute worst-case temporal penalty of open-loop hysteresis, the simulation utilizes this simplified reset-to-zero mathematical model. While advanced analytical feedforward models exist, forcing a physical return to the origin for every negative voltage step allows us to reliably benchmark the maximum timing overhead an algorithm might incur in the absence of complex software compensation.

### 3.4. Alignment Algorithms

We evaluate seven alignment algorithms deliberately selected to represent the complete methodological spectrum of optical optimization strategies. These seven methods span four distinct paradigms: (1) exhaustive grid searches (Reference Coarse, Reference Coarse+Fine) that establish fundamental ground-truth baselines; (2) dimensional reduction techniques (Cross Scan) that represent standard industry heuristics; (3) deterministic hill-climbing approaches (Fixed and Variable Gradient Ascent) that prioritize rapid, localized convergence; and (4) probabilistic machine-learning models (Local and Global Bayesian Optimization) designed to navigate noisy, hysteresis-distorted landscapes. By comparing this specific set, we can systematically evaluate the fundamental trade-offs between measurement efficiency, computational overhead, and ultimate alignment reliability. The algorithm descriptions below reflect the refined parameters and logic utilized during the simulation study. Specifically, the operational mechanics for each of the seven evaluated methods are detailed as follows:

- Reference Coarse Scan (Ref-Coarse):
  The reference coarse scan consists of an exhaustive raster scan with 4V voltage steps (corresponding to ∼0.5 μm spatial steps), followed by a move to the maximum detected power position. This establishes a baseline for global search, as detailed in Algorithm 1.

**Algorithm 1** Ref-Coarse  1: $P_{max}\leftarrow 0$, $\left(x_{max},y_{max}\right)\leftarrow \left(0,0\right)$  2: **for**
$V_{x}=0$ to 120 step 4 **do**  3:     **for**
$V_{y}=0$ to 120 step 4 **do**  4:         Move to $\left(V_{x},V_{y}\right)$  5:         P← Measure power  6:         **if**
$P>P_{max}$
**then**  7:            $P_{max}\leftarrow P$, $\left(x_{max},y_{max}\right)\leftarrow \left(V_{x},V_{y}\right)$  8:         **end if**  9:     **end for**10: **end for**11: Move to $\left(x_{max},y_{max}\right)$

**Figure 7:**
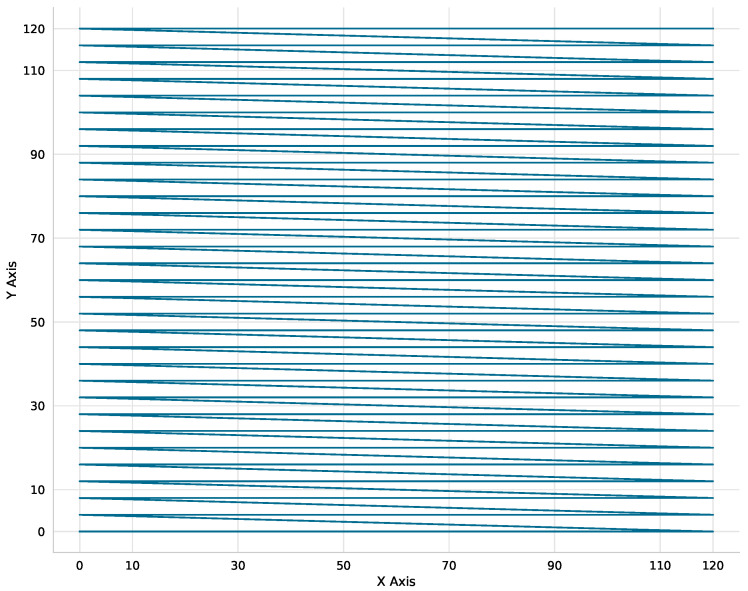
Schematic representation of the unidirectional (left-to-right) scanning strategy employed by the Reference Coarse algorithm to mitigate backlash and mechanical inconsistencies during grid exploration.

As illustrated in Figure 7, the Reference Coarse scan employs a strictly unidirectional (left-to-right) scanning strategy. This pattern is specifically designed to mitigate mechanical backlash and hysteresis inconsistencies during the exhaustive grid exploration.

- Reference Coarse + Fine Scan (Ref-Coarse+Fine):
  The Ref-Coarse+Fine scan utilizes a two-stage raster scan process. It begins with a coarse 6V-step scan (∼0.75 μm) which is followed by a fine 1V-step scan (∼0.125 μm) in a ±6 V (±0.75 μm) neighborhood around the coarse maximum. This method provides the highest accuracy at the cost of increased measurement points. The procedure is summarized in Algorithm 2.

**Algorithm 2** Ref-Coarse+Fine  1: Execute Algorithm 1 (with 6V step) to find $\left(x_{coarse},y_{coarse}\right)$  2: $P_{max}\leftarrow 0$, $\left(x_{max},y_{max}\right)\leftarrow \left(x_{coarse},y_{coarse}\right)$  3: **for**
$\Delta V_{x}=-6$ to +6 step 1 **do**  4:     **for**
$\Delta V_{y}=-6$ to +6 step 1 **do**  5:         $V_{x}\leftarrow x_{coarse}+\Delta V_{x}$  6:         $V_{y}\leftarrow y_{coarse}+\Delta V_{y}$  7:         Move to $\left(V_{x},V_{y}\right)$ (if within bounds)  8:         P← Measure power  9:         **if**
$P>P_{max}$
**then**10:            $P_{max}\leftarrow P$, $\left(x_{max},y_{max}\right)\leftarrow \left(V_{x},V_{y}\right)$11:         **end if**12:     **end for**13: **end for**14: Move to $\left(x_{max},y_{max}\right)$

- Cross Scan (Cross):
  The cross scan method is a two-stage dimensional reduction approach. The algorithm first performs a coarse cross-scan (4 V steps, ∼0.5 μm) along the X-axis at Y = 60 V (midrange), and then along the Y-axis at the optimal X position. It then refines the alignment with fine scans (1 V steps, ∼0.125 μm) in a ±4 V (±0.5 μm) neighborhood around the identified peak. This approach is highly efficient but remains sensitive to the specific landscape shape. Algorithm 3 outlines this two-stage process.

**Algorithm 3** Cross (Two-Stage)  1: *// Coarse Stage*  2: **for**
$V_{x}=0$ to 120 step 4 **do**  3:     Sample power at $\left(V_{x},60\right)$  4: **end for**  5: Find $x_{max}$ with highest power  6: **for**
$V_{y}=0$ to 120 step 4 **do**  7:     Sample power at $\left(x_{max},V_{y}\right)$  8: **end for**  9: Find $y_{max}$ with highest power10: *// Fine Refinement Stage*11: **for**
$V_{x}=x_{max}-4$ to $x_{max}+4$ step 1 **do**12:     Sample power at $\left(V_{x},y_{max}\right)$13: **end for**14: Find $x_{fine}$ with highest power15: **for**
$V_{y}=y_{max}-4$ to $y_{max}+4$ step 1 **do**16:     Sample power at $\left(x_{fine},V_{y}\right)$17: **end for**18: Move to observed maximum

- Local Bayesian Optimization (Local BO):
  The Local BO algorithm first performs a cross-scan to identify the region of interest, and then applies BO using a GP surrogate model in that local neighborhood (±20 V or ±2.5 μm). This hybrid approach combines the efficiency of initial cross-scan guidance with the intelligent exploration-exploitation balance of Bayesian methods. The complete routine is described in Algorithm 4.

**Algorithm 4** Local BO  1: *// Initial cross-scan (7 points per axis)*  2: **for**
$V_{x}\in \left\{0,20,40,60,80,100,120\right\}$
**do**  3:     Sample power at $\left(V_{x},60\right)$  4: **end for**  5: Find $x_{max}$ with highest power  6: **for**
$V_{y}\in \left\{0,20,40,60,80,100,120\right\}\setminus \left\{60\right\}$
**do**  7:     Sample power at $\left(x_{max},V_{y}\right)$  8: **end for**  9: $(x_{init},y_{init})\leftarrow$ best point from cross-scan (13 total points)10: Define search space: $x\in [x_{init}-20,x_{init}+20]$, $y\in [y_{init}-20,y_{init}+20]$11: Sample additional random points to reach 10 initial points in local region12: Fit Gaussian Process to observations13: **for** iteration =1 to 100 or until convergence **do**14:     Select next point via Expected Improvement15:     Measure power at selected point16:     Update Gaussian Process17:     **if** no improvement for 8 iterations **then**18:         Break19:     **end if**20: **end for**21: Move to observed maximum

The Expected Improvement (EI) acquisition function balances exploration and exploitation:
  (4) $$\mathrm{EI}\left(x\right)=E[\max\left(P\left(x\right)-P_{max},0\right)]$$
  where $P_{max}$ is the current best observed power.

- Global Bayesian Optimization (Global BO):
  The Global BO method searches the entire 120 V × 120 V space (15 μm × 15 μm) directly, without an initial cross-scan phase. This strategy provides the best exploration-exploitation balance for unknown landscapes through purely probabilistic sampling. Algorithm 5 details this approach.

**Algorithm 5** Global BO  1: Define search space: x∈[0,120], y∈[0,120]  2: Sample 8 Latin-Hypercube sampled points  3: Fit Gaussian Process to observations  4: **for** iteration =1 to 70 or until convergence **do**  5:     Select next point via Expected Improvement  6:     Measure power at selected point  7:     Update Gaussian Process  8:     **if** no improvement for 6 iterations **then**  9:         Break10:     **end if**11: **end for**12: Move to observed maximum

- Variable Gradient Ascent (Var-Grad):
  Var-Grad utilizes an adaptive gradient search logic to maximize alignment speed. The process begins with a cross-scan initialization (7 points per axis) to identify the initial signal vicinity, followed by iterative hill-climbing using finite-difference gradient estimates with a step size of h=2V (∼0.25 μm).
  The efficiency of this method is driven by an adaptive learning rate (α) that scales inversely with the measured power (*P*), governed by a gain factor k=250:
  (5) $$\alpha =\frac{k}{\max(P,10^{-12})}$$
  This mechanism allows the algorithm to take aggressive steps in low-power regions and gradually reduce the step size as it approaches the peak, enabling high-precision coupling that exploits the system 1 nm hardware resolution. While this approach achieved the fastest alignment time of 1.18 a.u. in HS-NH simulations, its dependency on local gradients makes it more susceptible to entrapment in local maxima compared to probabilistic methods. The pseudo-code for this adaptive approach is provided in Algorithm 6.

**Algorithm 6** Var-Grad  1: *// Initial cross-scan (7 points per axis)*  2: **for**
$V_{x}\in \left\{0,20,40,60,80,100,120\right\}$
**do**  3:     Sample power at $\left(V_{x},60\right)$  4: **end for**  5: Find $x_{max}$ with highest power  6: **for**
$V_{y}\in \left\{0,20,40,60,80,100,120\right\}\setminus \left\{60\right\}$
**do**  7:     Sample power at $\left(x_{max},V_{y}\right)$  8: **end for**  9: $(x_{0},y_{0})\leftarrow$ best point from cross-scan10: Measure $P_{0}$ at $\left(x_{0},y_{0}\right)$11: $\alpha \leftarrow 250/\max(P_{0},10^{-12})$
*(adaptive learning rate)*12: h←2
*(finite difference step)*13: **for** iteration =1 to 10 **do**14:     Measure $P_{1}$ at (x+h,y), $P_{2}$ at (x−h,y)15:     Measure $P_{3}$ at (x,y+h), $P_{4}$ at (x,y−h)16:     $\nabla_{x}\leftarrow \left(P_{1}-P_{2}\right)/\left(2h\right)$17:     $\nabla_{y}\leftarrow \left(P_{3}-P_{4}\right)/\left(2h\right)$18:     $\Delta x\leftarrow \alpha \nabla_{x}$, $\Delta y\leftarrow \alpha \nabla_{y}$19:     x←x+Δx, y←y+Δy20:     **if** |Δx|<0.5 and |Δy|<0.5 **then**21:         Break *(convergence)*22:     **end if**23: **end for**24: Move to best observed position

- Fixed Gradient Ascent (Fix-Grad):
  The Fix-Grad method is a traditional gradient search technique with fixed step sizes that halve upon observing no improvement. The procedure begins with a cross-scan (7 points per axis), and then iteratively explores neighbors in the cardinal directions (initial step 4 V or ∼0.5 μm, minimum 1 V or ∼0.125 μm) to identify the optimal ascent direction, as shown in Algorithm 7:

**Algorithm 7** Fix-Grad  1: *// Initial cross-scan (7 points per axis)*  2: **for**
$V_{x}\in \left\{0,20,40,60,80,100,120\right\}$
**do**  3:     Sample power at $\left(V_{x},60\right)$  4: **end for**  5: Find $x_{max}$ with highest power  6: **for**
$V_{y}\in \left\{0,20,40,60,80,100,120\right\}\setminus \left\{60\right\}$
**do**  7:     Sample power at $\left(x_{max},V_{y}\right)$  8: **end for**  9: $(x_{0},y_{0})\leftarrow$ best point from cross-scan10: Measure $P_{current}$ at $\left(x_{0},y_{0}\right)$11: δ←4
*(initial step size)*12: **while**
δ≥1
**do**13:     Measure power at (x+δ,y), (x−δ,y), (x,y+δ), (x,y−δ)14:     $(x^{\prime },y^{\prime },P^{\prime })\leftarrow$ best neighbor15:     **if** $P^{\prime }>P_{current}$ **then**16:         $\left(x,y,P_{current}\right)\leftarrow \left(x^{\prime },y^{\prime },P^{\prime }\right)$17:     **else**18:         δ←δ/2 *(reduce step size)*19:     **end if**20: **end while**21: Move to (x,y)


**Figure 8:**
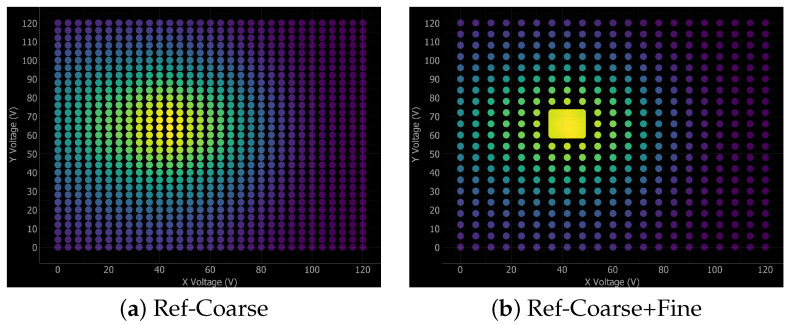

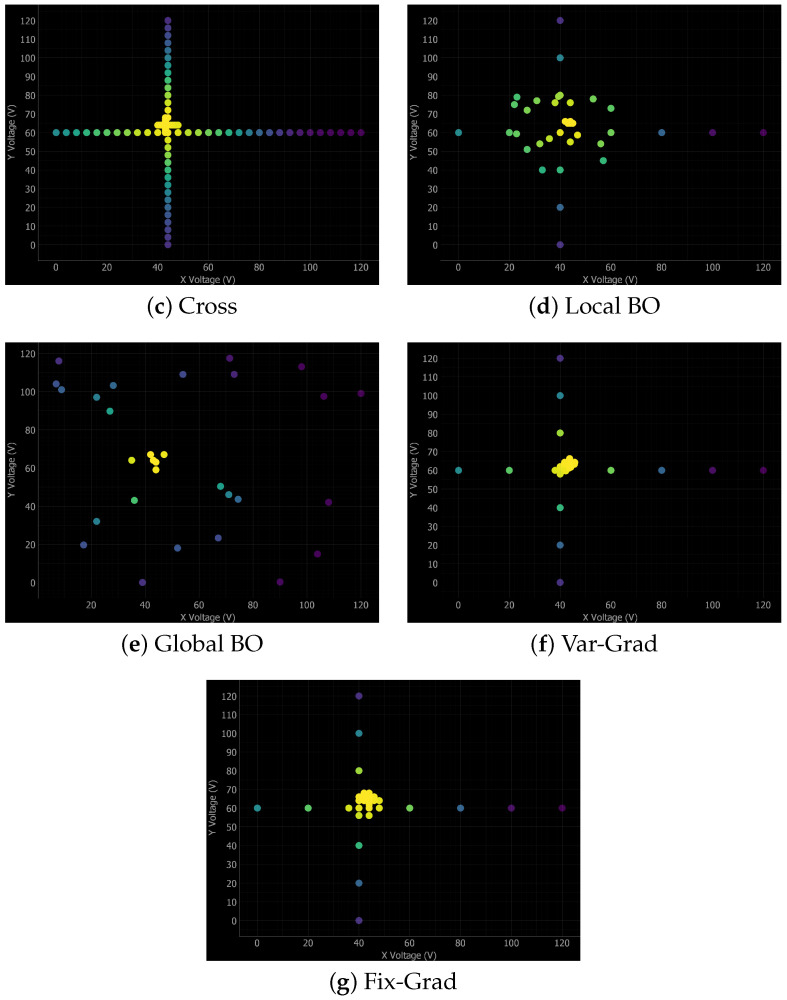
Comparison of all seven alignment algorithms on the same mock scan scenario. Each panel shows the search path of the algorithm overlaid as discrete colored measurement points, where the color intensity represents the measured optical power (ranging from dark purple for low power to bright yellow for the highest power). The figures demonstrate distinct search strategies: (**a**,**b**) exhaustive raster scanning, (**c**) dimensional reduction, (**d**,**e**) BO with intelligent sampling, (**f**,**g**) gradient-based hill climbing.

Figure 8 illustrates the application of all seven alignment algorithms to a representative mock scan scenario centered at (60 V, 60 V), providing a direct visual comparison of their respective search strategies. Each algorithm navigates the 120 V × 120 V operating range using a distinct optimization philosophy: Ref-Coarse and Ref-Coarse+Fine employ systematic raster scans; Cross scan utilizes dimensional reduction via orthogonal axis scanning; Local and Global BO implement intelligent sampling guided by Gaussian Process uncertainty; and Variable and Fix-Grad utilize hill-climbing techniques to track the local power gradient.

### 3.5. Performance Metrics and Statistical Analysis

Following best practices for algorithm comparison [18], we employ multiple evaluation approaches to ensure a robust assessment. First, for each algorithm on each problem instance, we record four individual metrics: a binary success indicator (defined as converging to ≥95% of the global maximum), the total number of measurement steps required, the total elapsed time including all movements and settling delays, and the final coupling accuracy (the achieved power divided by the global maximum power).

For the initial performance assessment, we compute a core set of aggregate quantitative indicators. The primary metric is the overall success rate, strictly defined as the percentage of alignment problems that were resolved successfully. Furthermore, we calculate central tendency metrics across all trials—specifically the mean number of alignment steps, total execution time, and final coupling accuracy. It should be noted that when reporting these average values, appropriate statistical caveats must be applied to account for the frequently non-normal distributions inherent in optimization data.

To rigorously account for this non-normal distribution of convergence steps, particularly as the number of test scenarios increases, we employ data profiles [40]. A data profile plots the fraction of problems solved as a function of the number of function evaluations (measurement steps):

(6) $$\rho_{s}\left(k\right)=\frac{1}{\left|P\right|}\left|\left\{p\in P:n_{s,p}\leq k\right\}\right|$$

where *P* is the set of test problems, *s* is a solver (algorithm), $n_{s,p}$ is the number of steps required by solver *s* on problem *p*, and *k* is the step budget. Data profiles provide a complete picture of algorithm performance by showing not just average behavior, but the full distribution of convergence speeds. An algorithm with a steep data profile quickly solves most problems, while a shallow profile indicates slower or less reliable convergence. In this work, data profiles are generated for the experimental results and for each specific simulation scenario (HS-H, HS-NH, LS-H, and LS-NH), enabling a comprehensive statistical comparison of algorithm performance across all operating conditions.

## 4. Results

### 4.1. Experimental Results

The algorithms are ranked based on a composite evaluation considering four critical metrics: (1) success rate, which is the primary criterion for production deployment; (2) average alignment time, reflecting throughput impact; (3) final accuracy, indicating optical coupling quality; and (4) average number of steps, representing measurement efficiency. Algorithms with 100% success rate are prioritized, then ranked by alignment time. Those with lower success rates are ranked lower regardless of speed, as unreliable alignment requires manual intervention and severely impacts production throughput.

Table 1 summarizes the performance of all seven algorithms across the eight experimental coupling scenarios.

**Figure 9:**
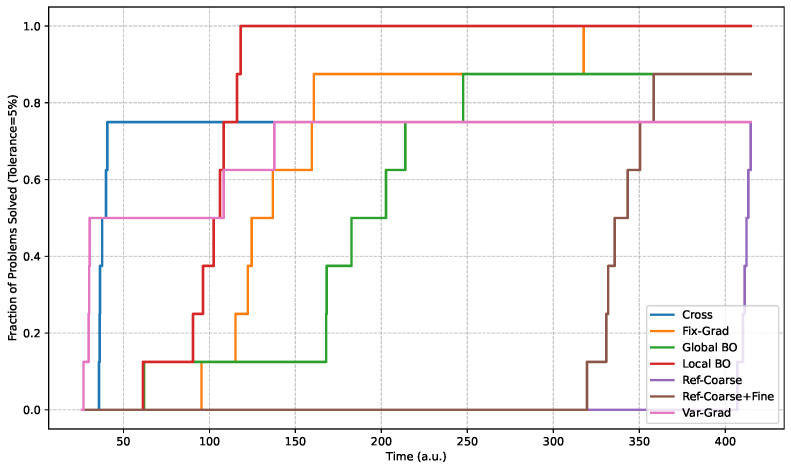
Data profile for experimental results. Local BO and Fix-Grad methods show steep profiles indicating rapid, reliable convergence to 100% success. Var-Grad converges fastest on easy problems but plateaus at 75%, while Ref-Coarse/Ref-Coarse+Fine require many measurements.

Experimental results indicate that the Local BO method achieves superior overall performance, delivering a 100% success rate, 98.24% accuracy, and rapid convergence (26.5 steps, 99.87 a.u.). This hybrid approach successfully balances exploration and exploitation via cross-scan initialization followed by Bayesian refinement. The Fix-Grad algorithm similarly achieves absolute reliability (100% success) alongside a marginally higher accuracy of 99.18%, although its systematic neighborhood exploration demands increased execution metrics (69.0 steps, 154.01 a.u.). In contrast, Global BO demonstrates strong general performance (97.60% accuracy, 37.4 steps) but yields an 87.5% success rate, indicating occasional difficulty navigating complex landscapes where optimal coupling regions remain distant from initial random samples. Furthermore, while the Ref-Coarse+Fine strategy provides adequate accuracy (98.31%), the exhaustive measurement requirement (600.3 steps, 334.11 a.u.) renders the approach entirely impractical for high-throughput manufacturing. The remaining techniques, that are Cross, Var-Grad, and Reference Coarse, exhibit heightened sensitivity to landscape topology, peaking at a 75% success rate. Notably, Var-Grad operates with the highest speed (22.5 steps, 59.28 a.u.) but lacks requisite dependability. Consequently, Local BO presents the optimal synthesis of speed, accuracy, and reliability for physical alignment tasks. Securing a 100% success rate is paramount for strict production environments to eliminate manual intervention and preserve critical operational throughput. Figure 9 shows the data profile for experimental results, plotting the fraction of problems solved as a function of total alignment time. The discrete, step-like appearance of these profiles is simply a consequence of the limited experimental sample size (N=8).

Following the methodology of Beiranvand et al. [18], data profile analysis elucidates comprehensive convergence behaviors that simple central tendency metrics obscure. Specifically, the Local BO profile exhibits a steep initial trajectory, achieving 100% success rate within approximately 30 steps. The Fix-Grad method similarly reaches absolute reliability by 80 steps, demonstrating a steady, albeit slower, progression. Conversely, algorithms such as Global BO, Var-Grad, and Cross exhibit rapid initial climbs but prematurely plateau at success rates of 87.5%, 75%, and 75%, respectively, indicating fundamental failure cases. Moreover, the exhaustive reference techniques Ref-Coarse and Ref-Coarse+Fine require hundreds of measurements merely to attain sub-optimal success rates between 75% and 87.5%. Ultimately, this statistical visualization demonstrates that while Var-Grad yields the lowest average step count (22.5), its early performance plateau and 25% failure rate render it significantly inferior to the robust Local BO and Fix-Grad algorithms for rigorous production deployment.

### 4.2. Simulation Results

Table 2 summarizes the average number of measurement steps required by each algorithm across the four simulation scenarios. Results are averaged over multiple mock files generated from the experimental data. Global BO requires the fewest steps (∼25), followed closely by Var-Grad (∼26) and Local BO (∼31). The intelligent sampling of Bayesian and Gradient methods reduces the measurement burden by over 20× compared to Ref-Coarse+Fine scanning (540 steps). The step counts are independent of the operating scenario, as they reflect algorithmic logic rather than timing. However, these step counts directly impact total time, which varies significantly across scenarios as shown next.

Table 3 shows total alignment time including movement and settling delays. Here, operating speed and hysteresis effects become apparent. The experimental data highlight the substantial throughput advantages of high-speed actuation. Transitioning from low-speed to high-speed regimes yields a roughly 24-fold acceleration for the Global BO algorithm, reducing alignment time from 53.06 to 2.24 a.u. under non-hysteretic conditions (HS-NH). However, managing uncompensated hysteresis introduces severe temporal overhead due to mandatory reset-to-zero motions, which inflates Global BO execution times by approximately 92% (from 2.24 to 4.29 a.u. in HS-H). Among the evaluated methods, Var-Grad achieves the absolute fastest alignment at 1.18 a.u. (HS-NH), although subsequent analysis reveals this speed incurs a significant reliability penalty. Conversely, Fix-Grad maintains a highly competitive balance, completing alignments in 1.44 a.u. (HS-NH) and 3.32 a.u. (HS-H) to offer a rapid, dependable alternative to Bayesian approaches.

Finally, to evaluate overall optimization quality and robustness, Table 4 details the achieved optical power as a percentage of the global maximum. Simulation results highlight the superior precision of the Fix-Grad method, which consistently achieves 99.4% of the global maximum power. Global BO and Local BO also perform excellently, achieving around 98% accuracy. Var-Grad shows improved accuracy compared to initial tests (98%), making it a competitive high-speed option.

**Figure 10:**
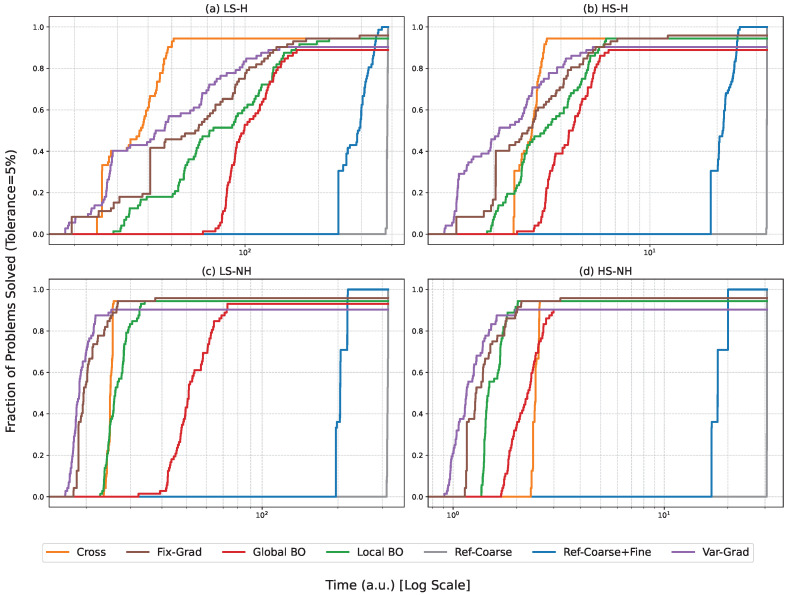
Data profiles for all four simulation scenarios showing the fraction of problems solved as a function of alignment time: (**a**) LS-H (Low-Speed with Hysteresis), (**b**) HS-H (High-Speed with Hysteresis), (**c**) LS-NH (Low-Speed without Hysteresis), and (**d**) HS-NH (High-Speed without Hysteresis). Note that the time axis is logarithmic to clearly visualize the significant speed advantage of active alignment methods (<5.1 a.u.) over exhaustive reference scans (>257.1 a.u.). Fix-Grad achieves the highest and most consistent reliability (95.8%) across all scenarios, while Global BO shows scenario-dependent reliability (88.9–93.1%) and Var-Grad shows the fastest initial convergence but lower final reliability (90.3%).

The performance of the seven investigated algorithms was evaluated across the 72 simulated test cases derived from experimental data. We utilize data profiles to represent the fraction of problems solved as a function of the total alignment time. A solved problem is defined as achieving a coupling power within 5% of the global maximum. Figure 10 illustrates these profiles across the four operating regimes. Figure 10b illustrates algorithm performance under realistic high-speed conditions with active hysteresis effects (HS-H). The Fix-Grad algorithm demonstrates the highest reliability (95.8%) across all 72 simulated test cases in the HS-H regime. Local BO and Global BO are competitive but exhibit lower final success rates (94.4% and 88.9%, respectively). Furthermore, while the Var-Grad method shows the fastest initial convergence for simple scenarios, solving a significant fraction of cases in under 2.6 a.u., it fails to achieve absolute reliability, plateauing at a 90.3% success rate due to its inherent sensitivity to hysteresis-induced local maxima. This highlights the robust advantage of the deterministic Fix-Grad approach, which maintains superior accuracy and dependability even when the mandatory reset-to-zero strategy introduces movement overhead to compensate for piezoelectric nonlinearities.

Comparing Figure 10b–d (HS-NH), the removal of hysteresis effects results in a leftward shift of the performance curves, reducing absolute alignment time by approximately 50–57% for gradient and Bayesian methods, consistent with the figures reported in Table 3. However, the relative performance ranking remains unchanged. This suggests that while hysteresis imposes a time penalty, the probabilistic nature of the Bayesian methods inherently manages these non-linearities more effectively than pure hill-climbing approaches.

In the Low-Speed regimes, the absolute time required for alignment increases significantly due to the lower slew rates and settling times. In the LS-H scenario, see Figure 10a, the Ref-Coarse and Ref-Coarse+Fine scans become impractical for volume production, requiring over 257.1 a.u. per die. The Cross provides a middle-ground baseline, consistently solving approximately 90% of the problems within a predictable time budget. However, like the gradient methods, it struggles with the highest-precision requirements. The Bayesian methods maintain their dominance in reliability, achieving the highest final success rates even as the absolute time scales upward.

Data profile analysis reveals distinct convergence behaviors across all evaluated scenarios. Local BO exhibits steep, consistent trajectories that secure high success rates within moderate step budgets, whereas Global BO demonstrates a similar but marginally slower initial ascent due to the absence of cross-scan initialization. Among gradient techniques, Var-Grad displays the steepest initial rise but suffers an early performance plateau, indicating fundamental failure cases on challenging coupling landscapes. Conversely, Fix-Grad maintains a steady progression toward maximum reliability, confirming its status as the most dependable gradient approach with a 100% experimental success rate. Furthermore, the one-dimensional Cross method prematurely plateaus near 75–80% success, while exhaustive reference techniques require an impractical hundreds of measurements to converge. Analyzed according to the statistical methodology of Beiranvand et al. [18], these profiles confirm that Local BO and Fix-Grad offer the best combination of speed and reliability across the operating conditions tested.

### 4.3. Algorithm Robustness

Table 5 quantifies the convergence reliability across all simulation scenarios, measuring the percentage of trials achieving ≥95% of global maximum power. The reliability analysis establishes Fix-Grad as the most robust optimization algorithm, achieving a consistent 95.8% success rate across all simulation scenarios. Global BO remains a strong contender, achieving 93.1% success in idealized (LS-NH) conditions and maintaining around 89% reliability in the presence of hysteresis. This represents a significant improvement over earlier baselines, although it does not yet match the consistency of the deterministic Fix-Grad method.

## 5. Discussion

### 5.1. Experimental Versus Simulation Performance

The experimental results in Table 1 provide a validation of algorithm performance on real PIC alignment tasks. The 100% success rates achieved by Local BO and Fix-Grad methods in experiments are particularly significant, as they demonstrate robustness across eight diverse coupling scenarios. This diversity stems from characterizations performed across seven different dies distributed to cover the full wafer surface as comprehensively as possible, spanning multiple reticle positions, supplemented by repeated measurements on the same die to account for environmental stability and system repeatability. By encompassing these variations in die location and characterization cycles, the results confirm that the proposed algorithms can generalize effectively across the typical process and placement variations encountered in high-volume wafer-level testing.

In terms of the interpretation of experimental rankings, results reflect the performance of algorithms with baseline parameters on a limited but highly realistic dataset of 8 coupling scenarios. Local BO achieved rank 1 due to its perfect 100% success rate combined with the fastest alignment time (99.87 a.u.) among the successful methods. Fix-Grad achieved rank 2, also with 100% success but requiring more time (154.01 a.u.). Global BO, despite strong performance metrics (97.60% accuracy), achieved only 87.5% success rate, placing it at rank 3. This lower success rate in the experimental phase reflects the conservative baseline parameters used at that stage, which had not yet been optimized for the specific characteristics of the Eclipse Dynamic system.

Following the experimental phase, an iterative improvement of the methods was conducted to refine the algorithm. The simulation study, which evaluated all the seven algorithms across 72 test scenarios (8 experiments × 9 spatial offsets), employed these improvements. This larger statistical sample and improved parameterization led to different performance rankings compared to the experimental results, revealing the true performance characteristics across diverse initial conditions.

The simulation framework reliably mimics real-world constraints. The RBF interpolation accurately captures landscape topologies, evidenced by the strong quantitative agreement between experimental data (e.g., Local BO experimental accuracy of 98.24%) and simulated outcomes (98.2–98.3%), indicating minimal uncertainty from the data processing and validating the fidelity of framework.. The difference in the success rates primarily reflects the evolution from baseline to changes of the algorithms and parameters, and the statistical power of the larger simulation dataset.

Given that the simulation results employ improved methods and parameters and a statistically rigorous evaluation across 72 scenarios, they provide the most reliable basis for production deployment recommendations. The experimental results validate the fundamental algorithmic approaches and confirm real-world viability, while the simulation results guide the selection of parameters and operating regimes.

### 5.2. Algorithm Selection Guidelines Across Operating Scenarios

The choice of an optimal alignment algorithm for the Eclipse Dynamic system depends on the operating speed capability and the effectiveness of hysteresis compensation. Comprehensive recommendations for each of the four evaluated scenarios are provided next, progressing from worst-case to ideal-case conditions.

#### 5.2.1. Scenario 1: LS-H (Low-Speed with Hysteresis)

This configuration establishes the baseline worst-case scenario, defined by conservative hardware actuation speeds coupled with uncompensated piezoelectric hysteresis. Under these conditions, the mandatory reset-to-zero maneuvers impose substantial temporal overhead, while the restricted slew rates further exacerbate the latency associated with each trajectory adjustment. Based on these stringent operating parameters, Fix-Grad emerges as the optimal choice for maximizing process yield (see Table 3 and Table 5). While the Var-Grad approach operates faster, its approximate 10% failure rate requires manual intervention, preventing any speed advantage in a production setting. Furthermore, the substantial temporal penalties imposed by mandatory reset-to-zero maneuvers disproportionately degrade the performance of Bayesian approaches in this slow-actuation regime, leaving Fix-Grad as the most robust and practical solution.

#### 5.2.2. Scenario 2: LS-NH (Low-Speed, No Hysteresis)

Algorithmic hysteresis compensation effectively eliminates the reset-to-zero overhead, although hardware actuation speeds remain inherently slow. This configuration successfully isolates the specific operational benefits of software-based compensation. With hysteresis effectively mitigated in software, alignment times improve across all methods (Table 3). Nevertheless, Fix-Grad maintains its operational dominance. Var-Grad provides a marginal speed advantage of roughly 2 a.u., but its lower reliability presents an unfavorable speed-versus-yield trade-off. Although the removal of physical reset maneuvers improves the relative viability of Bayesian methods compared to the LS-H scenario, their computational overhead keeps them slower than gradient-based alternatives, solidifying Fix-Grad as the definitively recommended strategy.

#### 5.2.3. Scenario 3: HS-H (High-Speed with Hysteresis)

Representing typical production conditions, this scenario pairs rapid hardware kinematics with uncompensated hysteresis. Evaluating algorithmic efficacy under these constraints is vital for industrial systems where implementing feedforward compensation remains impractical. In this realistic high-volume configuration, Fix-Grad is strongly recommended when alignment reliability is paramount, maintaining peak success rates while remaining highly competitive in execution speed. Var-Grad should be restricted to preliminary screening applications where occasional failures are an acceptable trade-off for rapid alignment. Notably, this scenario highlights a critical limitation of the Global BO approach: without cross-scan initialization, pure probabilistic exploration struggles to resolve the hysteresis-distorted landscape efficiently, resulting in both the lowest reliability and longest execution time among the optimization methods.

#### 5.2.4. Scenario 4: HS-NH (High-Speed No Hysteresis)

This ideal regime couples rapid hardware kinematics with effective feedforward hysteresis compensation [14,16]. Representing the gold standard for production deployment, this configuration highlights the ultimate capabilities of the algorithms. Fix-Grad is definitively recommended, delivering an optimal balance of high reliability and rapid alignment to effectively mitigate throughput bottlenecks [3]. While Var-Grad achieves the absolute fastest alignment time (approaching 1 a.u.), its persistent 9.7% failure rate remains a limiting factor for strict production environments. Conversely, despite requiring the fewest physical measurement steps, the substantial computational overhead of GP fitting severely inflates the temporal cost of the Global BO method, rendering it unsuitable for high-volume environments demanding both absolute reliability and sub-second speed.

### 5.3. Cross-Scenario Performance Analysis

A cross-scenario comparison reveals several critical insights. First, the Fix-Grad algorithm demonstrates exceptional robustness, consistently achieving 95.8% reliability across all speeds and hysteresis conditions. Conversely, Global BO is most sensitive to uncompensated hysteresis: in the LS regime, reliability drops from 93.1% to 88.9% (−4.2%), and in the HS regime, from 90.3% to 88.9% (−1.4%), while its alignment time inflates by 92%, rendering it problematic without active feedforward compensation.

Hardware kinematics and hysteresis mitigation drastically impact throughput. Transitioning from low-speed to high-speed regimes accelerates Fix-Grad alignment by 14.6× (from 20.95 to 1.44 a.u. without hysteresis) and 21.9× (from 72.47 to 3.32 a.u. with hysteresis). Managing hysteresis via the mandatory reset-to-zero strategy roughly doubles execution time across all algorithms (detailed in Table 3). Importantly, despite this severe temporal penalty, final positioning accuracy consistently exceeds 98%, validating the open-loop control architecture.

Finally, the data exposes a critical divergence between sample efficiency and true temporal efficiency. Although Global BO consistently requires the fewest physical measurements (24 steps), its substantial computational overhead for Gaussian Process updates results in a longer total execution time (2.24 a.u.) than Fix-Grad (1.44 a.u. across 34 steps). Consequently, sample efficiency alone is a misleading metric for evaluating high-volume production throughput.

### 5.4. Impact of Operating Speed

High-speed operation is essential for achieving production-viable throughput, as it drastically reduces per-die optical alignment time. Using the Fix-Grad algorithm as a benchmark, execution time decreases from 72.47 a.u. in the worst-case low-speed hysteretic regime (LS-H) to 20.95 a.u. in the low-speed non-hysteretic regime (LS-NH), 3.32 a.u. in the high-speed hysteretic regime (HS-H), and ultimately 1.44 a.u. in the ideal high-speed non-hysteretic regime (HS-NH). Transitioning from low-speed to high-speed operation therefore yields a 14.6-fold acceleration in the absence of hysteresis and an even larger 21.9-fold speedup when hysteresis is present.

The practical significance of this acceleration becomes clear at the wafer scale. For a standard 300 mm wafer containing 200 PIC dies, the total optical alignment burden rises to approximately 14,500 a.u. in the LS-H regime, which is clearly impractical for manufacturing. Under realistic high-speed operation with hysteresis (HS-H), this total is reduced to approximately 664 a.u., already representing a major improvement in throughput. In the ideal HS-NH regime, the total falls further to approximately 288 a.u. per wafer.

To relate these normalized values to real production conditions, we note that the ideal Fix-Grad alignment time of 1.44 a.u. is consistent with the sub-second per-die optical coupling times reported in state-of-the-art automated photonic test systems [10,11]. Under this interpretation, the HS-NH regime corresponds to full-wafer optical alignment in a matter of minutes, whereas the LS-H regime would translate into a multi-hour process devoted only to optical alignment. This difference clearly shows that high-speed actuation is not merely beneficial, but fundamentally necessary for meeting the throughput requirements of high-volume PIC and co-packaged optics manufacturing [1,3].

Overall, these results validate the importance of integrating high-speed piezoelectric drive electronics directly into the probe-card architecture. They also show that, when combined with an efficient search strategy such as Fix-Grad, the Eclipse Dynamic platform can achieve alignment times consistent with the fastest per-die coupling results reported in the recent literature [10,11].

### 5.5. Impact of Hysteresis

Hysteresis imposes a severe temporal penalty due to mandatory reset-to-zero motions, inflating high-speed execution times by 130% for the Fix-Grad algorithm (from 1.44 to 3.32 a.u.) and by 92% for the Global BO method (from 2.24 to 4.29 a.u.). Despite this near-halving of throughput, robust algorithms maintain highly stable alignment quality: Fix-Grad retains a constant 99.4% accuracy and 95.8% reliability regardless of hysteresis. Conversely, the Global BO approach experiences a reliability degradation from 90.3% to 88.9%, indicating that uncompensated hysteresis distorts the coupling landscape sufficiently to trap pure probabilistic exploration in local maxima. Ultimately, the business case for implementing feedforward hysteresis compensation, such as inverse P-I models [14,16], is highly compelling. Mitigating these nonlinearities effectively doubles system throughput and tester capacity, thoroughly justifying the initial software development investment.

### 5.6. Statistical Validity: Data Profiles Versus Averages

Data profile analysis (Figure 9 and Figure 10) confirms the above mentioned findings [18]. For instance, while Var-Grad exhibits a misleadingly low average step count (26.1), its data profile reveals a premature plateau at a 90.3% success rate, indicating fundamental algorithmic limitations rather than mere parameter mistuning. Conversely, the steep profile slopes of the Fix-Grad and Local BO algorithms demonstrate rapid, highly consistent convergence. From a practical manufacturing perspective, these profiles explicitly quantify worst-case performance and failure rates, directly informing throughput modeling by accounting for severe manual intervention overhead.

### 5.7. Comparison to State-of-the-Art

Contextualized against recent automated alignment benchmarks that report multi-second per-die execution times [10,11], the evaluated methods demonstrate highly competitive, sub-second performance. Operating entirely in an open-loop configuration, the Fix-Grad algorithm achieves a rapid 1.44 a.u. alignment time alongside 95.8% reliability and 99.4% accuracy. Furthermore, experimental validation of the Local BO method yields a 100% success rate and a 3–4× speedup (99.87 a.u.) over exhaustive reference scans. Crucially, these capabilities are realized without closed-loop position feedback, relying instead on computationally efficient algorithmic optimization, where even GP fitting runs in real-time on embedded systems and robust feedforward hysteresis compensation [14,33].

This performance underscores a critical innovation: deploying sophisticated algorithms to effectively compensate for simplified hardware mechanics. By eliminating the necessity for bulky external positioning equipment, this approach enables the cost-effective integration of optical alignment capabilities directly into the probe card. For high-volume PIC manufacturing [3,20], this integrated architecture provides a profound advantage. It inherently facilitates simultaneous multi-channel parallel testing, delivering multiplicative throughput enhancements that scale significantly beyond standard per-die speed improvements [5,12].

### 5.8. Limitations and Future Work

Several avenues warrant further investigation to advance automated wafer-level photonic testing. Future research will explore data-driven control architectures to further enhance system throughput. Specifically, replacing analytical hysteresis models with Long Short-Term Memory (LSTM) networks is expected to yield higher accuracy by better capturing deep, history-dependent piezoelectric behaviors. Concurrently, Reinforcement Learning (RL) agents offer the potential to adaptively counteract complex mechanical wear and drift over the lifespan of the probe card, outperforming static algorithms for dynamic fine-stage optimization.

Additionally, expanding these methods to multi-fiber array alignments will require strategies to balance peak loopback transmission with channel uniformity and crosstalk limits across the array. Because the FAU moves as a rigid block, optimizing a single channel can induce misalignment on peripheral channels due to planar tilt. Utilizing the differential Y1/Y2 piezoelectric actuators for precise rotational tilt compensation will be essential in navigating these multi-channel compromises. Finally, standardizing chip layouts, such as fixing input/output port offsets and reference waveguide lengths across different PIC designs, would significantly narrow the spatial bounding required for search algorithms, accelerating the cross-scan initialization phase for both Bayesian and Gradient methodologies. To mitigate the initialization challenges observed in Bayesian methods, historical wafer data can also be leveraged to generate predictive spatial probability maps, drastically reducing initial search areas. Furthermore, online learning and advanced sensor data imputation techniques [44] must be developed to maintain alignment robustness against real-time probe drift and intermittent signal degradation.

Transitioning these methodologies to high-volume manufacturing demands broader experimental validation across diverse foundries, device architectures, and multi-fiber arrays. In such high-dimensional optimization landscapes, the algorithmic hierarchy may shift favorably toward Bayesian surrogate models [5,11]. Crucially, these sub-second alignment strategies must be seamlessly integrated into double-sided test cells to meet the aggressive throughput demands of CPO production flows [1,3]. Finally, coupling operational alignment metrics with digital twin frameworks and transformer-based sensor placement [45,46] will enable robust structural health monitoring, facilitating predictive maintenance and extending probe card lifespans in demanding industrial environments.

## 6. Conclusions

This paper presented a comprehensive evaluation of alignment optimization techniques for wafer-level photonic integrated circuit testing, utilizing the Technoprobe Eclipse Dynamic probe card system. Through robust experimental validation and extensive simulations spanning diverse operating regimes, incorporating varying actuation speeds and hysteresis conditions, this study systematically benchmarked seven alignment algorithms. The results unequivocally establish the Fix-Grad algorithm as the optimal solution for high-volume manufacturing. Across all evaluated conditions, this approach demonstrated exceptional robustness, consistently delivering a 95.8% reliability rate and 99.4% alignment accuracy.

Transitioning to high-speed actuator drive electronics is essential for production viability, providing a 14.6–21.9× throughput improvement over low-speed operation (Table 3). In the ideal high-speed, hysteresis-compensated regime, the Fix-Grad algorithm achieved a rapid alignment time of 1.44 a.u. per die. While uncompensated hysteresis approximately doubles this execution time, algorithmic mitigation strategies successfully preserve final measurement quality without requiring complex closed-loop positioning feedback. Alternative methods presented specific trade-offs—the Var-Grad technique offered the fastest alignment (1.18 a.u.) but suffered a higher failure rate of 9.7%, rendering it suitable only for non-critical screening. Furthermore, despite high sample efficiency, BO approaches exhibited excessive computational overhead and heightened sensitivity to hysteresis, limiting their viability for strict production environments.

From a production perspective, data profile analysis confirms that the high reliability of the Fix-Grad method limits manual operator intervention to just 4.2% of tested dies. For a standard 300 mm wafer containing 200 dies, this integrated architecture facilitates complete optical characterization in approximately 288 to 664 a.u., depending on the extent of active hysteresis compensation. By achieving highly reliable optical alignments entirely through open-loop control, this system matches or exceeds the performance metrics reported in recent literature [10,11]. Crucially, embedding this capability directly into the probe card eliminates the need for bulky external positioning stages, significantly reducing overall equipment footprint and enabling the parallel multi-channel testing required for emerging CPO architectures [1,3].

Future research will focus on advancing data-driven control strategies to further enhance system throughput and absolute accuracy. Key initiatives include implementing Long Short-Term Memory networks to replace analytical models for history-dependent hysteresis compensation, alongside deploying Reinforcement Learning agents to adaptively counteract complex mechanical irregularities. Additionally, leveraging historical wafer data to generate predictive spatial heatmaps will heavily optimize search initialization parameters. Expanding these methodologies to accommodate simultaneous multi-fiber array alignments and fully integrating the resulting automated flows into advanced test cells will be essential to meet the escalating manufacturing demands of next-generation silicon photonics.

## Figures and Tables

**Table 1:** Experimental alignment results across 8 coupling scenarios. Algorithms ranked by combined performance considering success rate, time, accuracy, and measurement efficiency. Results obtained with baseline algorithm parameters.

Rank	Method	Success Rate	Avg Time (a.u.)	Avg Accuracy (%)	Avg Steps
1	Local BO	100%	99.87	98.24	26.5
2	Fix-Grad	100%	154.01	99.18	69.0
3	Global BO	87.5%	175.19	97.60	37.4
4	Ref-Coarse+Fine	87.5%	334.11	98.31	600.3
5	Cross	75.0%	36.63	96.84	80.0
6	Var-Grad	75.0%	59.28	95.99	22.5
7	Ref-Coarse	75.0%	410.90	96.81	961.0

**Table 2:** Average number of measurement steps.

Algorithm	HS-H	HS-NH	LS-H	LS-NH
Ref-Coarse	961.0	961.0	961.0	961.0
Ref-Coarse+Fine	539.6	539.6	539.6	539.6
Cross Scan	75.6	75.6	75.6	75.6
Local BO	30.7	31.3	31.2	30.9
Global BO	24.6	24.7	23.9	24.6
Var-Grad	26.1	26.1	26.1	26.1
Fix-Grad	34.4	34.4	34.4	34.4

**Table 3:** Average total alignment time (a.u.).

Algorithm	HS-H	HS-NH	LS-H	LS-NH
Ref-Coarse	33.39	30.64	382.60	315.01
Ref-Coarse+Fine	21.26	18.10	286.07	205.53
Cross Scan	2.88	2.44	35.42	24.88
Local BO	3.60	1.54	85.29	26.63
Global BO	4.29	2.24	103.26	53.06
Var-Grad	2.39	1.18	51.00	18.97
Fix-Grad	3.32	1.44	72.47	20.95

**Table 4:** Average final power coupling (% of global maximum).

Algorithm	HS-H	HS-NH	LS-H	LS-NH
Ref-Coarse	99.9	99.9	99.9	99.9
Ref-Coarse+Fine	100.0	100.0	100.0	100.0
Cross Scan	98.2	98.2	98.2	98.2
Local BO	98.2	98.3	98.3	98.3
Global BO	98.2	98.0	97.9	98.4
Var-Grad	98.0	98.0	98.0	98.0
Fix-Grad	99.4	99.4	99.4	99.4

**Table 5:** Convergence reliability of the alignment algorithms (defined as the percentage of trials reaching ≥95% of optimal power).

Algorithm	HS-H	HS-NH	LS-H	LS-NH
Ref-Coarse	100.0	100.0	100.0	100.0
Ref-Coarse+Fine	100.0	100.0	100.0	100.0
Cross Scan	94.4	94.4	94.4	94.4
Local BO	94.4	94.4	94.4	94.4
Global BO	88.9	90.3	88.9	93.1
Var-Grad	90.3	90.3	90.3	90.3
Fix-Grad	95.8	95.8	95.8	95.8

## Data Availability

The data presented in this study are available on request from the corresponding author.
